# Facts from Correspondents

**Published:** 1846-08

**Authors:** 


### FACTS FROM CORRESPONDENTS.

---

### A SHOWER OF MAGGOTS.

Communicated by *M. H. Fee*, M.D., of Leatherwood, Ia.

A strange phenomenon occurred in this town on the 18th inst., in the form of insects, commonly called "*skippers*," falling from a cloud accompanied by rain. The truth of the thing can be substantiated by at least six persons in the vicinity of my residence, whose attention was called to the circumstance by myself. The cloud arose in the west about 1 o'clock, P.M. The general course of Leatherwood creek at this place, and for a mile or two below, is west, so that the cloud in approaching the town touched many parts of the creek. A few minutes before the rain commenced falling here, I heard the noise of a water spout, or what Dr. Lardner terms, I believe, "partial attraction," about two miles below this, in the direction of the general course of the creek. The character of the noise, I cannot describe; all I can say is it was a *peculiar* noise, differing entirely from the sound of wind at a distance, or of murmuring thunder. I may very appropriately say of it, what Dr. Laennec says of the peculiarity of sounds in the thorax, in auscultation, "known only to the practised ear." Now, the only reasonable conclusion I can come to with regard to the manner in which these insects got their elevation in the air, is, that the "water spout" in its course up the creek, had passed over some stagnant ponds, or puddles, in which the dead carcass of some animal had been lying partially covered by water for perhaps months, or at least for such a length of time as was necessary for the generation of maggots, which were drawn up with the surrounding water.

The appearance of the maggots corresponded in every particular with that of the "skipper" found in bacon. They varied in length from one-eighth to one-third of an inch; were blunt at the hinder end, sharp at the head, and covered, as far back as the head extended, with short, black, bristly hair. After the shower was over and the sun came out, the heat caused them to double themselves and skip about from place to place, in search of shade.

June 22d, 1846.

Dr. Fee records an interesting phenomenon, but one by no means unprecedented in the annals of meteorological science. Many well authenticated instances are on record of animals precipitated from the upper regions to the earth during storms. A few years ago a number of small fish were picked up on one of the roads leading out of Louisville, two miles from the river, whence they had been carried by a gust of wind, the preceding night. We conversed with a gentleman in Jeffersonville, sometime since, who, in a hurricane which swept across the river just above the falls, saw fish going up with the ascending column of water. Frogs have been known to be carried up in a similar manner, and to fall at considerable distances in numbers. In tornadoes it is well known that even heavy bodies, as the branches of trees, are often elevated to great heights, and transported many miles. The surprising circumstance in Dr. Fee's case is that the storm should have come across the "skippers;" but that hardly equals the Tennessee wonder, in which the clouds poured down "flesh and blood." Our friend, Dr. Troost, the distinguished professor of Chemistry and Mineralogy in the University of Nashville, published shortly after this strange occurrence a learned paper on the subject of it, in which he gave an account of a great variety of bodies which have been seen to fall to the earth in different countries and at different periods, having been previously elevated into the air by the force of the winds. We regret that we have mislaid the interesting article and are therefore unable to copy some of its details.

